# SPIN90 Phosphorylation Modulates Spine Structure and Synaptic Function

**DOI:** 10.1371/journal.pone.0054276

**Published:** 2013-01-14

**Authors:** In Ha Cho, Dae Hwan Kim, Min-Jung Lee, Jeomil Bae, Kun Ho Lee, Woo Keun Song

**Affiliations:** 1 Bio Imaging and Cell Dynamics Center, School of Life Sciences, Gwangju Institute of Science and Technology, Gwangju, Korea; 2 Department of Marine Life Science, Chosun University, Gwangju, Korea; Univ. Kentucky, United States of America

## Abstract

The correct rearrangement of postsynaptic components in dendritic spines is important for driving changes of spine structure and synaptic function. SPIN90 plays an essential role in many cellular processes including actin polymerization, endocytosis, growth cone formation and dendritic spine morphogenesis. Here, we demonstrate that SPIN90, which is a binding partner of PSD95 and Shank in spines, is targeted to synapses and leads to enhanced synaptic activity in neurons. We show, using *in vitro* and *in vivo* kinase assays, that SPIN90 is tyrosine phosphorylated by Src kinase. SPIN90 that was tyrosine-phosphorylated by Src was targeted to dendritic spines in cultured hippocampal neurons. Moreover, a SPIN90 phospho-deficient mutant was unable to accumulate at dendritic spines whereas SPIN90 WT and a phospho-mimicking mutant were localized at spines and bound PSD95 and Shank with increased efficiency. Consistent with these findings, hippocampal neurons that overexpressed SPIN90 WT or a phospho-mimicking mutant had enlarged spine heads, leading to enhanced postsynaptic function in terms of both amplitude and frequency. Together, our findings show that SPIN90 modulates synaptic activity in neurons as a result of its phosphorylation.

## Introduction

Dendritic spines are the postsynaptic components of excitatory synapses that mediate the transmission of information between neurons. The examination of evoked changes in dendritic spine morphology could be an effective means of elucidating the mechanisms governing synaptic activity. Furthermore, because spines play a key role in synaptic transmission, investigation of how they are formed and regulated might provide insight into the structural basis of learning and memory [1].

Dendritic spine structure and function are regulated by the correct targeting and maintenance of structural and signaling molecules [2], [3]. For example, PAR-1 (Partitioning-Defective 1) modulates dendritic spine morphogenesis by phosphorylating the PSD (Postsynaptic density) scaffolding protein, PSD95 [4]. SAP97 is a scaffolding protein implicated in the synaptic targeting of NMDA- and AMPA-type glutamate receptors [5]. It modulates synaptic plasticity by controlling the surface distribution of glutamate receptors. In contrast, inappropriate targeting of glutamate receptors reduces synaptic transmission and results in impaired synaptic function. PSD95 is targeted to synapses by its palmitoylation [6], [7], and results in recruitment of AMPA receptors to the synapses [8]. However, much still remains to be elucidated about the molecular mechanisms underlying the translocation of synaptic proteins to spines.

Dendritic spine morphogenesis is mediated by various actin-regulating proteins that are involved in modulating the actin cytoskeleton [9]. GRK5 (G protein-coupled receptor kinase 5) is essential for F-actin bundling through its F-actin-binding domains, which leads to maturation of dendritic spines [10], while PICK1 regulates spine shrinkage by inhibiting Arp2/3 activity [11]. How actin-regulating proteins function and interact with actin and/or other PSD proteins so altering the structural and functional plasticity of spines is therefore of great interest.

SPIN90 was initially identified as a Nck binding partner [12], and is now known to regulate actin polymerization through its interactions with Arp2/3, N-WASP and actin [13]. It participates in many actin-related cellular processes. In particular, DIP (mDia-interacting protein), another name for SPIN90, is involved in stress fiber formation downstream of the Rho-mDia pathway [14]. SPIN90 was found to participate in Rac-induced membrane ruffling [15], indicating that it is an important actin regulator. It is strongly expressed in the brain, especially in the cerebellum, cortex and hippocampus [16]. During development, SPIN90 increases and accumulates within dendritic spines, where it contributes to dendritic spine morphology by interacting with PSD95 and Shank. However, its role in synaptic activity has not previously been investigated.

In the present study, we show that tyrosine phosphorylation of SPIN90 is crucial for its synaptic targeting, and that phosphorylated SPIN90 mediates spine enlargement, thereby enhancing synaptic function. A SPIN90 phospho-mimicking mutant, but not SPIN90 phospho-deficient mutants, enhanced postsynaptic function as detected by increased miniature EPSC amplitude and frequency. This is the first report demonstrating the mechanism of SPIN90 synaptic targeting and its significance in synaptic function.

## Results

### Phosphorylated SPIN90 Locates to Spines

**Figure 1:**
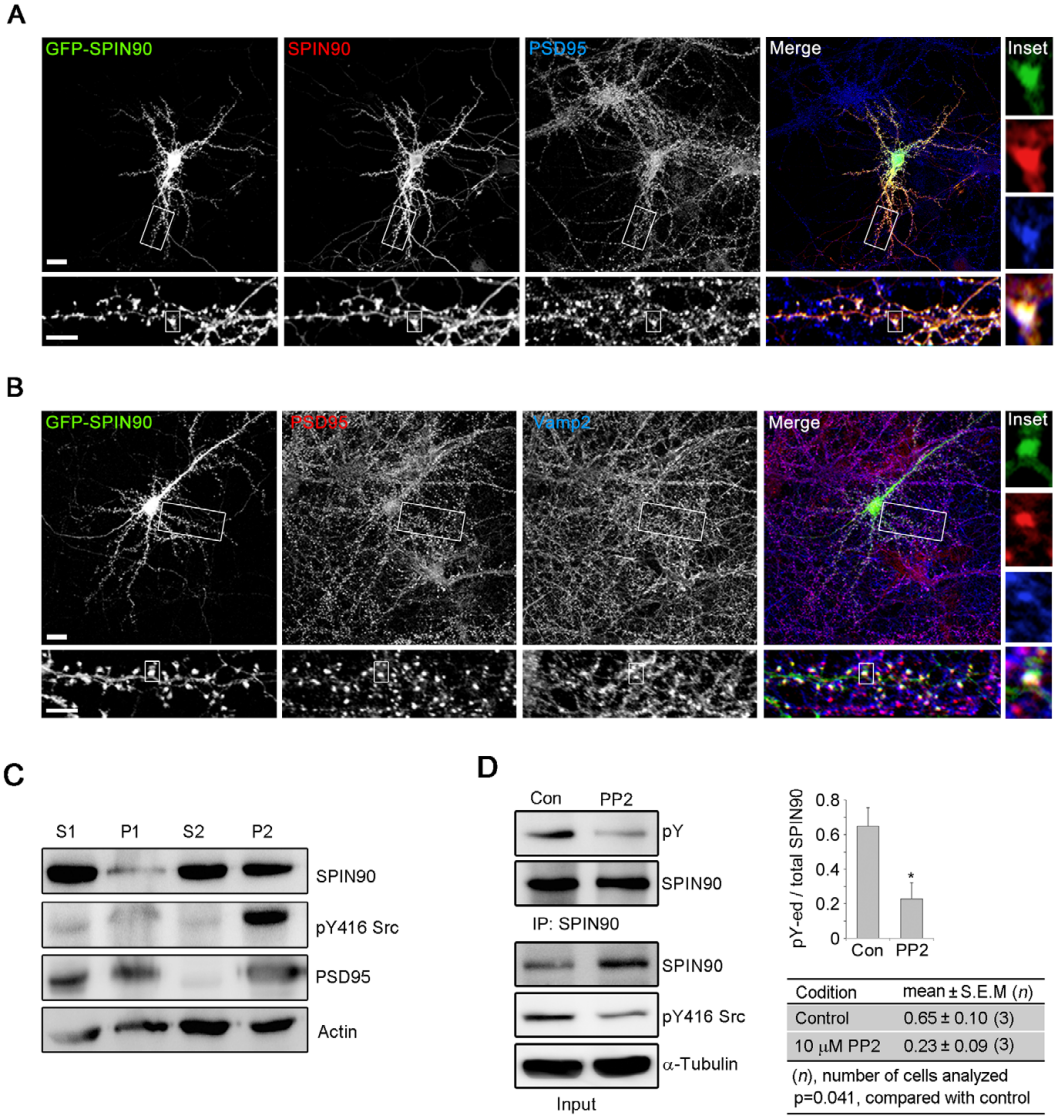
Phosphorylated SPIN90 is localized to spines in neurons. (**A**,**B**) Rat hippocampal neurons transfected with GFP-SPIN90 at DIV 10-12 were fixed at DIV 19–21 and labeled with the indicated antibodies. (**C**) Mouse brain lysates were fractionated to generate crude synaptosomal (P2) and cytosolic fractions (S2), and equal amount of protein was examined by immunoblotting. (**D**) Rat cortical neurons (DIV 18) were incubated with or without PP2 (10 µM for 30 min), after which SPIN90 phosphorylation was assessed using anti-phosphotyrosine antibody (pY).

It is known that SPIN90 is located within dendritic spines and interacts with PSD proteins [16], [17]. To assess the mechanism underlying its synaptic targeting, we performed immunocytochemical assays on GFP-SPIN90-transfected hippocampal neurons. Anti-SPIN90 antibody clearly stained GFP-SPIN90 spots, as revealed by an exact match between GFP signals and signals generated by immunostaining with anti-SPIN90 antibody (Fig. 1A). Moreover GFP-SPIN90 accumulated at spines in association with the postsynaptic marker, PSD95, and adjacent to the presynaptic marker, Vamp2 (Fig. 1B) [16].

It was previously shown that DIP (referred to as SPIN90) could be phosphorylated by Src kinase [14]. This suggested that SPIN90 might be a substrate of Src kinase in synaptic regions. To test the association of SPIN90 and Src, we first fractionated synaptosomes. SPIN90 was found equally in the crude synaptosomal fraction (P2) and the cytosolic fraction (S2), whereas PSD95 was particularly enriched in P2, as shown in previous work [18]. Both SPIN90 and active Src detected by anti-pY416 Src antibody were found in P2 (Fig. 1C). Moreover, treatment of neurons with PP2, an inhibitor of Src kinase [19], reduced the SPIN90 phosphorylation significantly (Fig. 1D). Taken together, these data suggested that SPIN90 is a substrate for Src kinase in neurons.

### SPIN90 is Tyrosine-phosphorylated by Src Kinase

**Figure 2:**
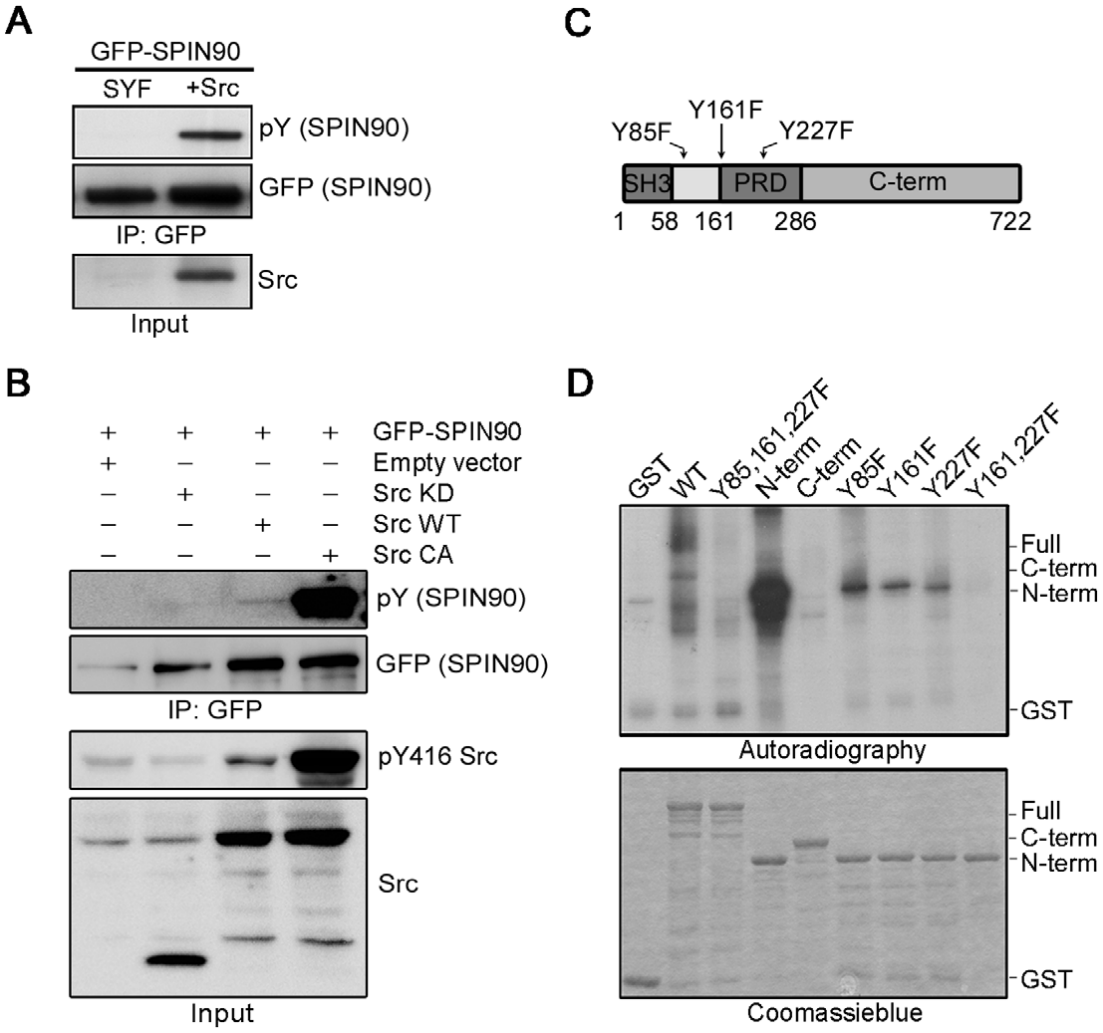
SPIN90 is phosphorylated by Src kinase. (**A**) Src-catalyzed SPIN90 phosphorylation was tested in SYF (deficient in Src, Yes, Fyn) and c-Src recovery cells in SYF cell lines. (**B**) COS7 cells were transfected with empty vector, Src KD, Src WT or Src CA together with GFP-SPIN90 and immunoprecipitated with anti-GFP antibody. (**C**) Schematic diagram of SPIN90 showing the positions of tyrosine phosphorylated residues. The indicated domains are as follows: SH3, Src homology 3; PRD, proline-rich domain. (**D**) *In vitro* phosphorylation of GST-SPIN90 proteins. Bacterially overexpressed GST, GST-SPIN90 domains or mutant proteins were incubated with recombinant Src (5 U) for 10 min at 30°C. Phosphorylated products were revealed by autoradiography.

For *in vivo* Src kinase assays, GFP-SPIN90 was overexpressed in SYF cells (Src, Yes, Fyn deficient fibroblasts) and in c-Src recovery cells from SYF cell lines. As predicted, phosphorylated SPIN90 was detected in the c-Src recovery cells (Fig. 2A). In accord with this, when GFP-SPIN90 was co-transfected with Src CA (a constitutively active form of Src; SrcY527F) into COS7 cells, it was found in a highly tyrosine-phosphorylated form. In contrast, phosphorylated GFP-SPIN90 was not found in COS7 cells co-transfected with Src KD (a kinase dead form of Src; Src251) (Fig. 2B). *In vitro* kinase assays identified three potential tyrosine phosphorylation sites (Y85, Y161 and Y227) in the N-terminus of SPIN90 and demonstrated that the triple mutation completely abolished SPIN90 phosphorylation (Fig. 2C,D).

### Phosphorylated SPIN90 Locates in Spines

**Figure 3:**
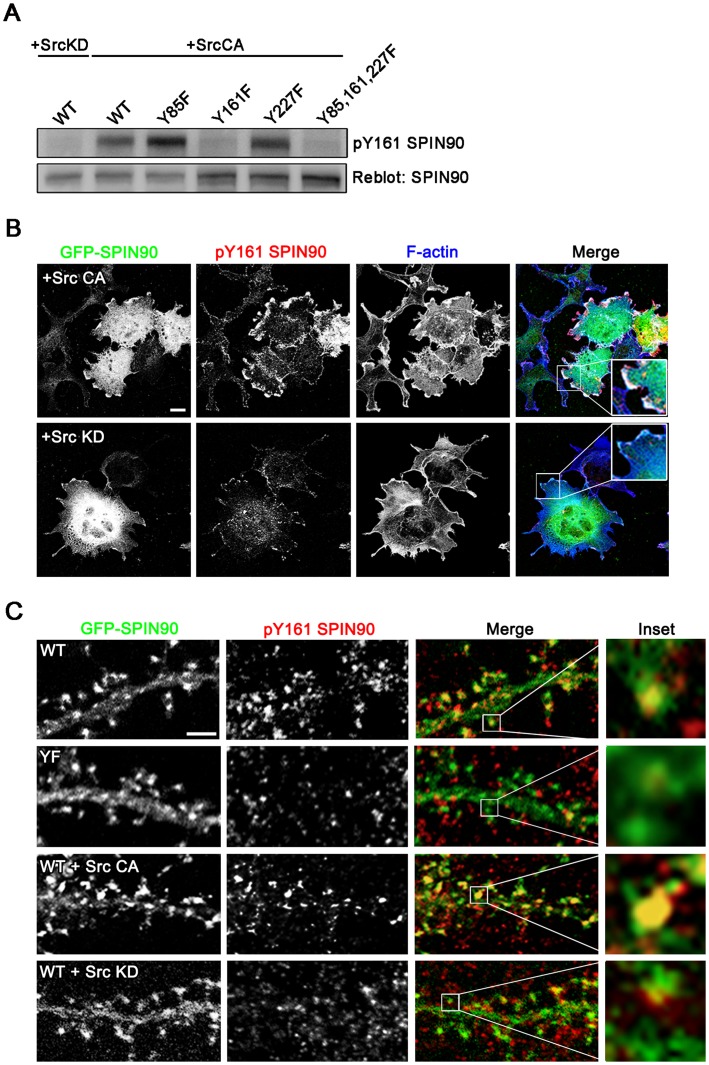
Phosphorylated SPIN90 locates in spines. (**A**) Cell lysates from COS7 cells transfected with various SPIN90 constructs (GFP-SPIN90 WT, Y85F, Y161F, Y227F and Y85, 161, 227F) together with Src KD or Src CA were analyzed by immunoblot assay using anti-SPIN90 or anti-pY161 SPIN90 phosphospecific antibody. (**B,C**) COS7 cells and hippocampal neurons at DIV 19 were cotransfected with GFP-SPIN90 WT, Src CA or Src KD and then immunostained with anti-pY161 antibody. Phalloidin (blue) was used to stain F-actin and reveal dendritic spines.

To further characterize the phosphorylation of SPIN90, we immunized rabbits with three peptides mimicking the phosphorylated tyrosines in the three tyrosine phosphorylation sites of SPIN90 (Y85, Y161or Y227). We were able to produce phospho-specific antibody (anti-pY161 antibody) against phospho-tyrosine 161 of SPIN90 but not against pY85 and pY227 of SPIN90. To confirm the specificity of the anti-pY161 antibody, we co-infected COS7 cells with GFP-SPIN90 WT or phospho-deficient mutants (tyrosines substituted with phenylalanine at positions Y85, Y161 or Y227), together with Src CA or Src KD. The corresponding cell lysates were analyzed by immunoblot assay with anti-SPIN90 antibody to measure the expression of the transfected SPIN90 constructs, and this confirmed that all the proteins were expressed at similar levels (Fig. 3A). Anti-pY161 antibody detected SPIN90 WT and two of the SPIN90 mutant proteins (Y85F and Y227F) but not SPIN90 Y161F or the SPIN90 triple-mutated protein (Y85, 161, 227F) (Fig. 3A), confirming that the anti-pY161 antibody specifically recognized SPIN90 phosphorylated at tyrosine residue 161. Parallel experiments demonstrated that anti-pY161 antibody also detected GFP-SPIN90 co-expressed with Src CA in membrane ruffles of COS7 cells (Fig. 3B).

Next we tested whether SPIN90 in spines is phosphorylated by Src kinase. Because anti-pY161antibody does not detect endogenous SPIN90, GFP-SPIN90 WT or YF were overexpressed in hippocampal neurons. Anti-pY161 detected GFP-SPIN90 WT, but not GFP-SPIN90 YF, in spines (Fig. 3C). In accord with this, when GFP-SPIN90 was co-transfected with Src CA, but not Src KD, was detected by anti-pY161 antibody strongly. Taken together, these data show that SPIN90 in spines is tyrosine phosphorylated.

**Figure 4:**
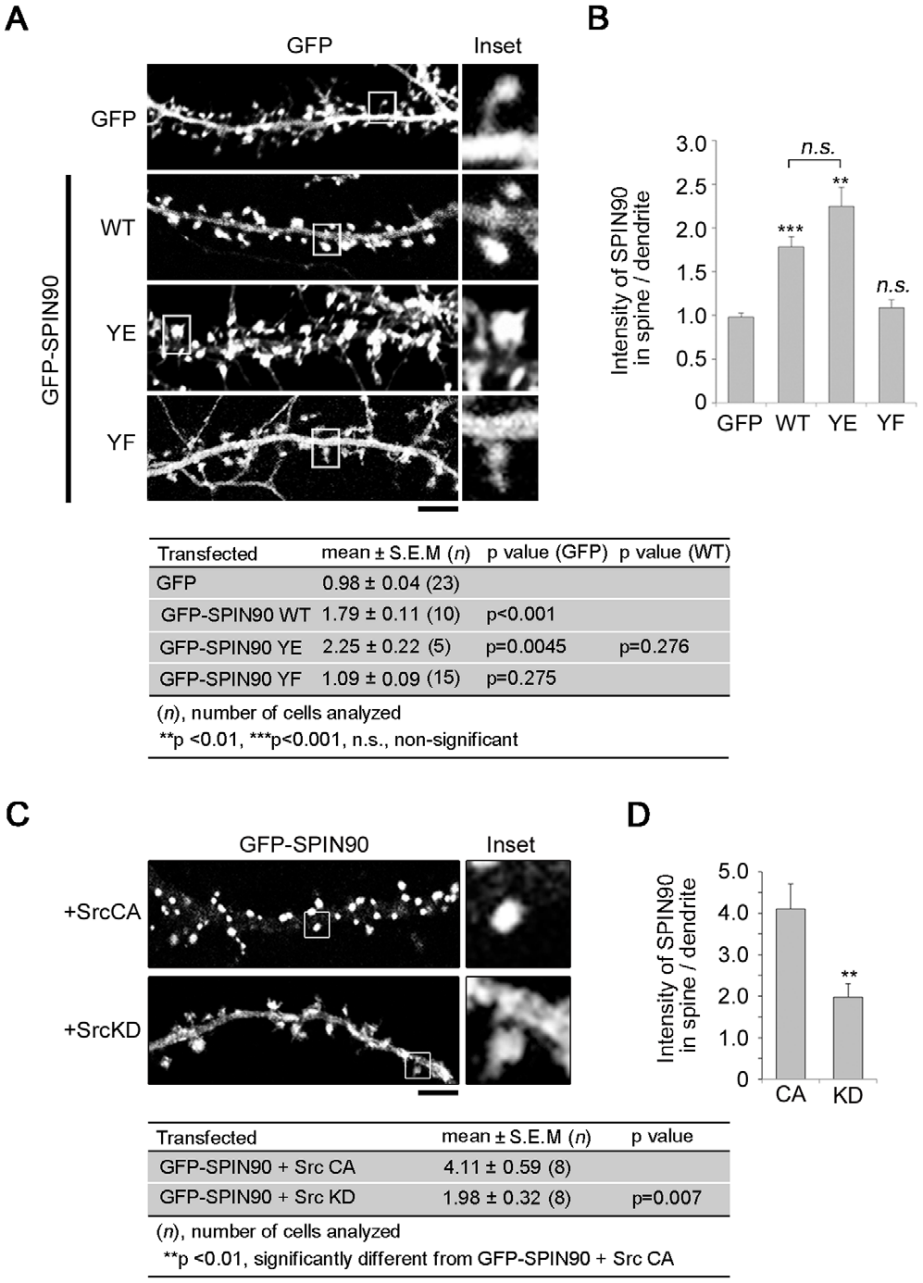
SPIN90 phosphorylation induces its synaptic targeting. (**A**) Immunofluorescence analysis revealing the distribution of transfected GFP empty vector, and GFP-SPIN90 WT, YE and YF within hippocampal neurons (DIV 19–21). (**B**) For quantification, averaged fluorescence intensities of GFP-SPIN90 in spine vs. the dendritic shaft were analyzed as described in **Materials and Methods**. (**C**) Hippocampal neurons cotransfected with GFP-SPIN90 WT and Src CA or Src KD were fixed at DIV19. (**D**) Averaged fluorescence intensities of GFP-SPIN90 in spines vs. dendritic shafts.

When we overexpressed GFP empty vector or GFP-SPIN90 WT, YE or YF in hippocampal neurons, SPIN90 WT and YE accumulated within dendritic spines, but GFP empty vector and SPIN90 YF were evenly distributed between spines and shafts (Fig. 4A,B). In addition, GFP-SPIN90 co-transfected with Src CA, but not Src KD, readily accumulated in spines (Fig. 4C,D). These results indicate that phosphorylation of SPIN90 by Src is essential for its synaptic targeting.

### SPIN90 Phosphorylation Modulates its Association with the PSD Scaffolding Proteins, PSD95 and Shank1b

**Figure 5:**
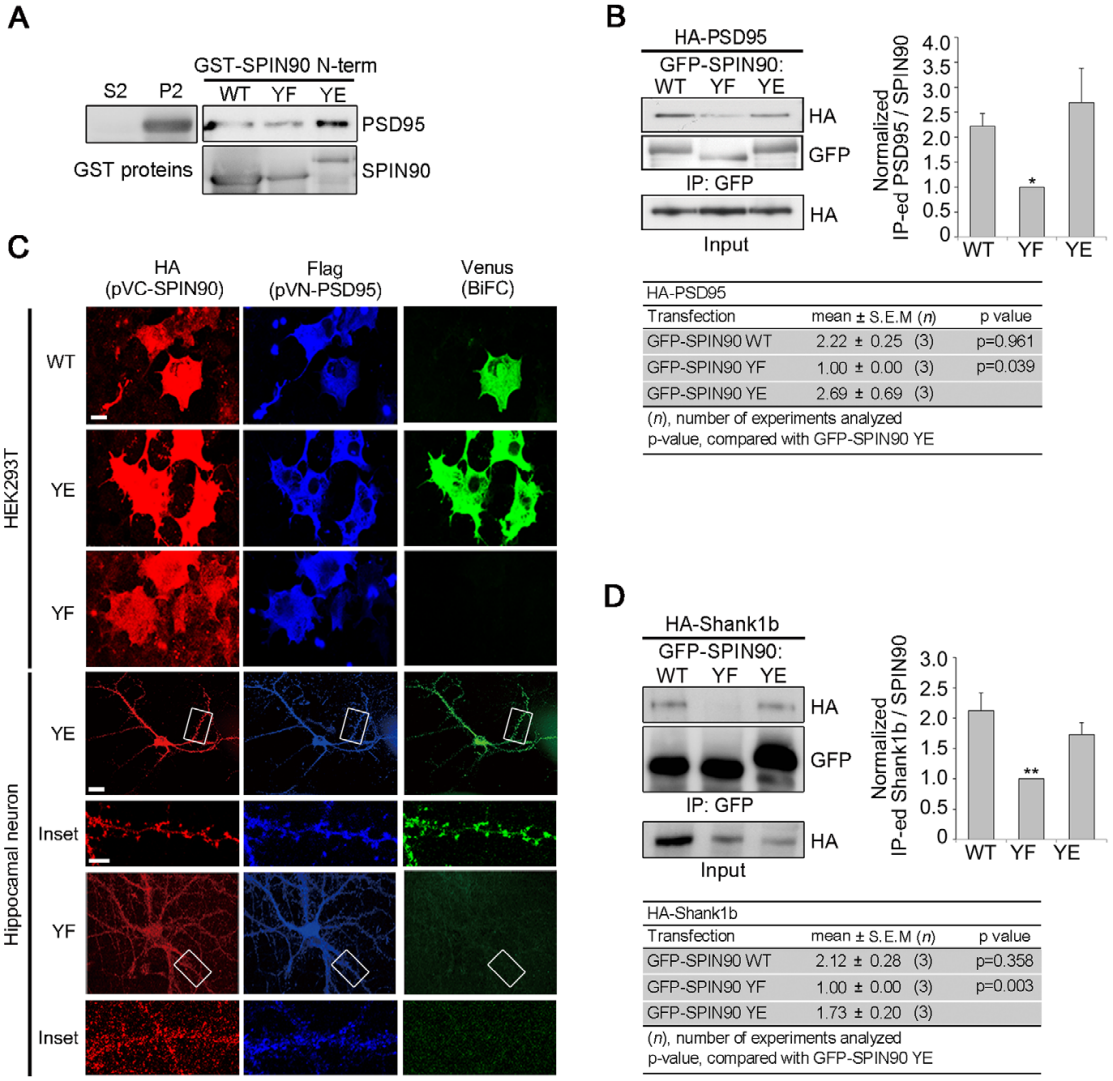
SPIN90 phosphorylation modulates its association with PSD scaffolding proteins. (**A**) *In vitro* binding assays carried out with GST-SPIN90 WT, YE or YF agarose beads and lysates from crude synaptosomal fraction. The bound proteins were verified using anti-PSD95 and anti-SPIN90 antibodies. (**B**) The interaction between SPIN90 and PSD95 was analyzed in HEK293T cells co-transfected with HA-PSD95 plus GFP-SPIN90 WT, YF or YE. (**C**) In BiFC assays, HEK293T cells and cultured hippocampal neurons co-expressing VN-PSD95 and VC-SPIN90 WT, VC-SPIN90 YE or VC-SPIN90 YF were monitored for the venus signal (green). Cells were also immunostained with anti-Flag antibody to detect VN-PSD95, and with anti-HA antibody to detect VC-SPIN90. Scale bars, 10 µm in HEK293T cells and 20 µm in neurons. (**D**) The interaction between SPIN90 and Shank1b was analyzed in HEK293T cells co-transfected with HA-Shank1b plus GFP-SPIN90 WT, YF or YE.

The finding that SPIN90 phosphorylation affects its synaptic localization led us to examine whether the interaction of SPIN90 with PSD scaffolding proteins was dependent on its phosphorylation. GST pull-down assays with GST-SPIN90 YE and extracts of mouse P2 fraction revealed interaction between the SPIN90 phospho-mimicking protein and PSD95. On the other hand, when bacterially expressed SPIN90 WT or YF was incubated with the P2 extract, the interaction between SPIN90 and PSD95 was found to be markedly reduced (Fig. 5A). To characterize the effect of SPIN90 phosphorylation on the interaction with PSD95 *in vivo*, HA-PSD95 and the various GFP-SPIN90 mutants were expressed in HEK293T cells; immunoprecipitation assays demonstrated a dramatically reduced interaction of SPIN90 YF with PSD95, compared with SPIN90 WT or YE (Fig. 5B). In addition, using the BiFC method, SPIN90 WT or YE co-expressed with PSD95 gave strong venus signals (green) in HEK293T cells and hippocampal neurons (Fig. 5C), showing that their interaction is highly dependent on SPIN90 phosphorylation. Interestingly, the interaction between SPIN90 and Shank1b was also dependent on SPIN90 phosphorylation (Fig. 5D). Since PSD95 and Shank are crucial components of postsynaptic regions and essential for the formation and maintenance of dendritic spines, the phosphorylation-dependent SPIN90 binding to scaffolding proteins indicates that SPIN90 phosphorylation is important for modulating dendritic spine morphology.

### SPIN90 Phosphorylation Regulates Spine Size and Synaptic Function

**Figure 6:**
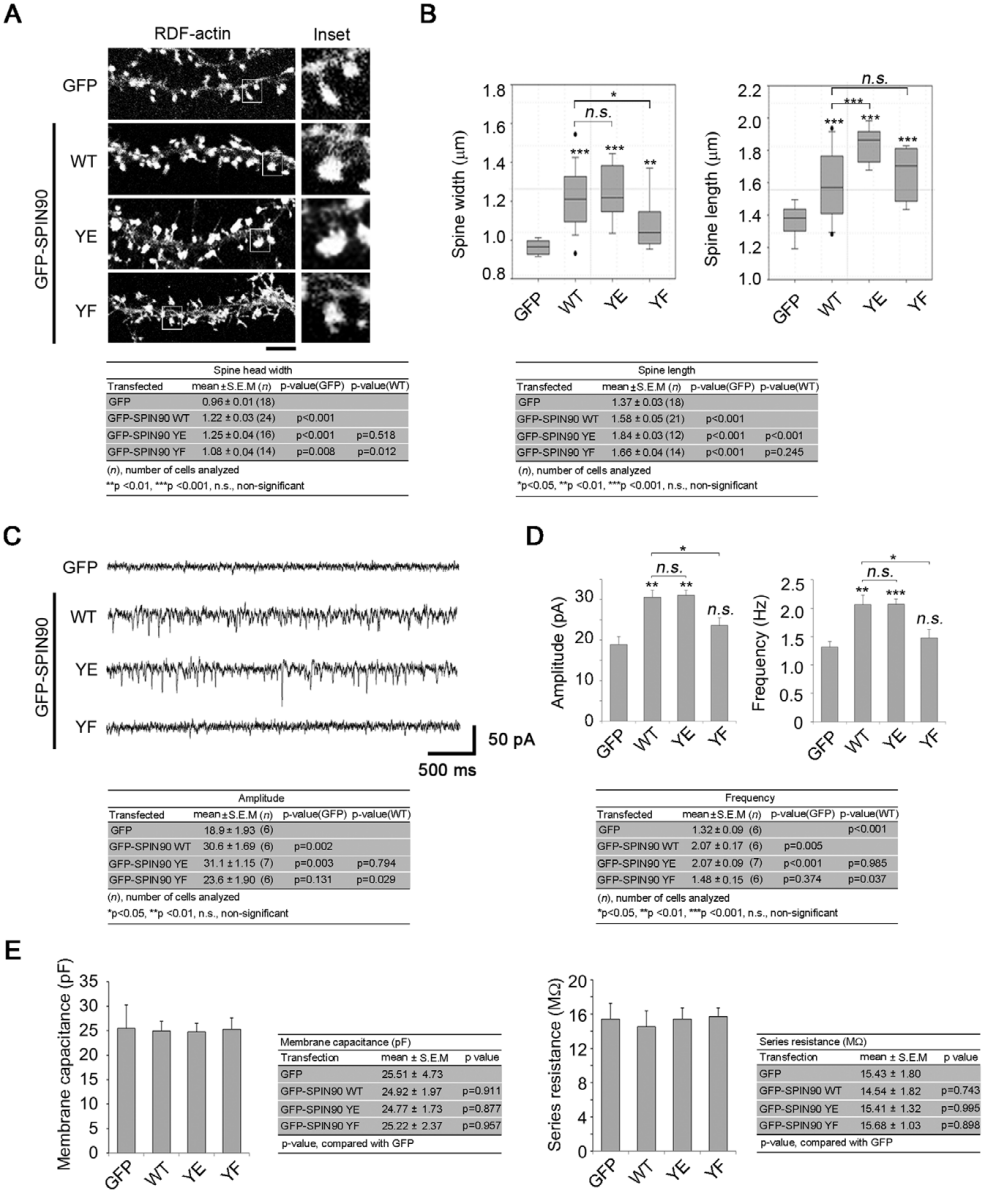
SPIN90 dephosphorylation impairs synaptic function. (**A**) Cultured hippocampal neurons were co-transfected with GFP empty vector, GFP-SPIN90 WT, YE or YF plus RDF-actin at DIV 10–12, and spine morphology was analyzed at DIV 19–21. RDF-actin was used to visualize dendritic spines. (**B**) Spine head width and length in neurons expressing SPIN90 YE or YF were analyzed using Box and whisker plots. (**C**) mEPSCs were recorded from neurons transfected with GFP only or with GFP-SPIN90 WT, YF or YE. (**D**) Shown are averaged mEPSC amplitudes and frequencies. (**E**) Membrane capacitance (pF) and series resistance (MΩ) were compensated and measured by Axopatch 200 B after whole-cell membrane penetration during all of the voltage-clamped experiments. Shown are normalized values of membrane capacitance and series resistance of GFP only or SPIN90 YE, YF, and WT transfected neurons.

An earlier report that the interaction of SPIN90 with Shank and PSD95 within dendritic spines promoted an increase of spine size [17] led us to test whether SPIN90 phosphorylation plays a role in spine morphogenesis. Cultured hippocampal neurons were transfected with GFP empty vector or GFP-SPIN90 WT, YE or YF at DIV 10–12 and spine head widths and lengths were analyzed at DIV 19–21. Co-transfection of RDF-actin enabled the spine morphology to be visualized. Spine head width was markedly increased in SPIN90 WT and YE-transfected cells, as compared with SPIN90 YF- and GFP-transfected cells, and spine length was slightly greater in SPIN90 YE-transfected cells than in SPIN90 WT- and YF-transfected cells (Fig. 6A,B). This suggests that SPIN90 phosphorylation contributes to the regulation of dendritic spine enlargement.

It is known that the morphology of dendritic spines is strongly related to synaptic function [9]. We therefore used the whole-cell patch clamp technique to investigate the role of SPIN90 phosphorylation at synapses. Hippocampal neurons at DIV 10–12 were transfected with GFP-SPIN90 WT, YE or YF, or GFP alone. To assess postsynaptic activity, spontaneous mEPSCs (holding potential: −70 mV) were recorded (Fig. 6C,D). Overexpression of GFP-SPIN90 WT or YE led to significant increases in mEPSC amplitude and frequency, as compared to neurons expressing GFP alone. On the other hand, no significant change was observed in the amplitude and frequency of SPIN90 YF-transfected neurons. Normalized values of membrane capacitance (C_m_), which is measured in terms of the area of the membrane, and series resistance (R_s_) did not show significant change among GFP control and GFP-SPIN90-variant transfected neurons (Fig. 6E). This showed that patched cells in each experimental group shared similar characteristics including cell size. These findings suggest that SPIN90 phosphorylation contributes to the modulation of synaptic activity by regulating spine morphology.

## Discussion

It has been suggested that the morphology of dendritic spines is directly related to synaptic function [20]. Therefore, it is of central importance to understand the molecular mechanisms that modulate dendritic spine morphology. In addition, the fact that a number of neurological disorders, including Down’s syndrome, autism and Alzheimer’s disease, are closely associated with altered spine morphology and number highlights the importance of dendritic spine morphology [21], [22], [23], [24]. Hence understanding the cellular processes that control dendritic spine morphology may provide insight into postsynaptic changes of synaptic plasticity.

It is well known that there are many different proteins in PSD regions and that their selective synaptic targeting is crucial for synaptic transmission. For example, the targeting of proteasomes, which is mediated by interaction with CaMKII, is important for the activity-dependent degradation of poly-ubiquitinated proteins in spines [25], [26]. Also, selective transport of mRNA is essential for dendritic spine stabilization [27]. In accord with this, the synaptic targeting of the RNA-binding protein, TLS, which participates in mRNA sorting, affects dendritic spine morphology [28]. In addition, it has been shown that many actin-interacting proteins such as cortactin, WAVE, drebrin and neurabin regulate actin structure, leading to changes of dendritic spine morphology [2], [29], [30], [31]. Therefore, it is essential to characterize the mechanism of synaptic targeting of PSD proteins and their functions in spines.

Our earlier studies pointed to an important role for SPIN90 in the formation and maintenance of dendritic spines. However, the mechanism of SPIN90 synaptic targeting and the role of SPIN90 in synaptic activity were unclear. Here we demonstrated that SPIN90 phosphorylation by Src kinase is directly related to its synaptic targeting. Overexpressed SPIN90 YE accumulated in spines and enhanced dendritic spine head width, leading to increased synaptic activity.

Scaffolding proteins such as PSD95 and Shank determine the synaptic localization of their binding partners such as cortactin and GKAP [32]. An important observation in the present study was that the interaction of SPIN90 with PSD95 and Shank1b was enhanced by tyrosine phosphorylation of the PRD regions of SPIN90, and that the phospho-deficient SPIN90 YF mutant was not accumulated at synapses. This indicated that phosphorylation of the PRD regions of SPIN90 is essential for synaptic localization. On the other hand, earlier work suggested that the SPIN90 C-terminal domain was important for synaptic targeting [16]. The SPIN90 C-terminus is able to bind F-actin, G-actin and the Arp2/3 complex, and its interaction with the Arp2/3 complex promotes Arp2/3-mediated actin polymerization [13]. Thus its C-terminus may help to target SPIN90 to dendritic spines, which are enriched in F-actin, while its tyrosine phosphorylation likely facilitates its synaptic targeting and promotes its interaction with PSD proteins. For example, the interaction of the SPIN90 C-terminus with the Arp2/3 complex may promote actin polymerization within dendritic spines, leading to the formation of mature spines.

Phosphorylated SPIN90 (WT and YE) increased spine head width relative to SPIN90 YF. SPAR, a PSD95 binding partner, also increased spine head width, and deletion of the PSD95 binding domain of SPAR abolished its effect on spine morphology, suggesting that its binding with PSD95 is essential for the control of spine morphology [33], [34]. In addition, binding of IRSp53 to PSD95 is required for its synaptic targeting [35]. Accordingly, it is likely that the interaction of phosphorylated SPIN90 with PSD95 and Shank is essential for spine head enlargement. It is well known that morphological changes of dendritic spines lead to changes in synaptic strength. As predicted, neurons transfected with constructs encoding phosphorylated SPIN90 showed increased synaptic activity. Consequently, these findings suggest that SPIN90 plays a crucial role in the regulation of dendritic spine morphogenesis and subsequent synaptic plasticity.

In contrast to spine head width, spine length was increased by SPIN90 YF as well as SPIN90 WT and YE. Earlier studies demonstrated that the Arp2/3 complex likely regulates both dendritic spine head enlargement and spine elongation [2], [11], [20], [36]. In particular, a regulatory effect of Arp2/3 on spine length has been reported. The interaction of cortactin with Arp2/3 induces spine elongation [2], and PICK1 inhibits Arp2/3 activity and so decreases spine length [11]. The increase of spine length in SPIN90-overexpressing neurons thus may be due to activation of the Arp2/3 complex through its C-terminus rather than to tyrosine phosphorylation of the SPIN90 N-terminus. This question requires further elucidation.

Physiological and morphological modifications occur in conjunction with long-term synaptic alterations such as LTP and LTD [37], and it is generally accepted that long-term synaptic alterations are accompanied by spine shrinkage and AMPA receptor endocytosis, although this is controversial [38], [39], [40]. SPIN90 is known to regulate clathrin-mediated endocytosis in association with endocytotic proteins such as syndapin and dynamin I [41], [42]. It has been proposed, for example, that SPIN90 (DIP) deficiency affects LTD induction by modulating AMPA receptor endocytosis [43]. Previous studies have shown that chemical LTP causes a redistribution of SPIN90 from dendritic shaft to spine [16]. Taken together, these findings suggest SPIN90 participates in the functional modification of synaptic strength, including LTP and/or LTD. Hence, it will be important to elucidate the molecular mechanisms by which SPIN90 modulates synaptic plasticity.

In summary, phosphorylated SPIN90 is enriched in dendritic spines and co-localizes with postsynaptic proteins. Tyrosine phosphorylation of SPIN90 by Src kinase is crucial for its synaptic targeting, and increases its affinity for PSD scaffolding proteins. We conclude that synaptic targeting of phosphorylated SPIN90 is a key requirement for enhanced synaptic development as well as basal synaptic activity.

## Materials and Methods

### Ethics Statement

All animal experiments were approved by the Gwangju Institute of Science and Technology Animal Care and Use Committee (the permit number: GIST-2010–14).

### Antibodies and Reagents

Polyclonal anti-SPIN90 serum was described previously [44]. Mouse monoclonal antibodies were used against PSD95 (Abcam, ABR), phosphotyrosine, HA (Millipore), GFP (Invitrogen), α-tubulin, Flag (Sigma) and Src (Cell Signaling). Rabbit polyclonal antibodies were used against Vamp2 (ABR) and pY416-Src (Cell Signaling). Goat polyclonal antibody was used against actin (Santa Cruz). Horseradish peroxidase-conjugated anti-mouse, anti-rabbit and anti-goat secondary antibodies were purchased from the Jackson Laboratory. Alexa Fluor 488-, 555-, 594- and 647-conjugated donkey anti-rabbit IgG, and donkey anti-mouse IgG were from Molecular Probes. PP2 (4-Amino-5-(4-chlorophenyl)-7-(t-butyl)pyrazolo[3,4-d]pyrimidine), an inhibitor of Src kinase, was acquired from Tocris.

### Plasmids

SPIN90 was inserted into pEGFP vector (BD Clontech) or pGEX-4T-1 (Amersham Biosciences), after which the site-directed mutants Y85, 161, 227F and Y85, 161, 227E were generated from GFP-SPIN90 using a QuikChange TM site-directed mutagenesis kit (Stratagene). The expression vector pLHCX was purchased from Clontech. pLHCX-Src was used as template for the Src Y527F (Src CA) and Src 251 (Src KD) constructs. HA-Shank1b and HA-PSD95 were kindly provided by Dr. Okabe (Tokyo Medical and Dental University, Japan).

### Cell Culture, Transfection and Imaging

HEK293T, COS7 and SYF were maintained in Dulbecco’s modified Eagle’s medium supplemented with 10% fetal bovine serum. For primary neuronal cultures, hippocampal and cortical neurons were collected from E18–19 rats and cultured in neurobasal medium (Invitrogen) supplemented with B27 (Invitrogen) and 2 mM GlutaMAX (Invitrogen). For biochemical experiments we used cortical neurons because they provide enough cell lysate to conduct biochemical analyses (e.g., immunoprecipitation assays). Hippocampal neurons were used for cell imaging experiments. Cells were plated on poly-D-lysine-coated coverslips at a density of 4×10^5^ cells/60 mm dish. Neurons were transfected at DIV 11–12 using a modified calcium phosphate precipitation method. To determine the effects of SPIN90 phosphorylation and accumulation within dendritic spines and excitatory synapses, neurons were visualized at DIV 18–21. Cells were fixed with 4% paraformaldehyde and sucrose in PBS and permeabilized with 0.25% Triton X-100. They were then incubated with the appropriate primary antibodies followed by Alexa Fluor-conjugated secondary antibodies. F-actin was stained with Alexa Fluor 555-coupled phalloidin (Invitrogen) for 90 min at room temperature. Images were then obtained using a Fluoview FV 1000 confocal laser-scanning microscope equipped with 100x and 60x oil-immersion objectives and capable of additional 3x to 4x zoom.

### Bimolecular Fluorescence Complementation (BiFC) Assays

To visualize protein interactions, BiFC assays were performed; these are based on the premise that two non-fluorescent fragments of a fluorescent protein can form a fluorescent complex, and that association between the fragments can be facilitated [45]. SPIN90 and its mutants (SPIN90 Y85, 161, 227E and SPIN90 Y85, 161, 227F) were subcloned into pBiFC-VC155, and PSD95 was subcloned into pBiFC-VN173. After transfection of hippocampal neurons and HEK293T cell lines with these BiFC constructs, the cells were fixed and imaged using an FV1000 confocal microscope.

### Co-immunoprecipitation and Western Blot Analysis

Cells were briefly washed with cold PBS and extracted for 1 h at 4°C in modified radioactive immunoprecipitation assay buffer (50 mM Tris-HCl, pH 7.4, 150 mM NaCl, 1% Nonidet P-40 (NP-40), 0.25% sodium deoxycholate, 10 mM NaF, and 1 mM Na_3_VO_4_) supplemented with protease inhibitors. The extracts were then clarified by centrifugation for 10 min at 13,000 rpm, and the protein concentrations in the supernatants were determined using the Bradford assay (Bio-Rad Laboratories). The resultant extracts were incubated with primary antibodies overnight at 4°C, followed by 4 h incubation with protein A/G sepharose beads (GE Healthcare). The resultant immunoprecipitates were washed extensively with extraction buffer, separated on SDS-PAGE and transferred to PVDF membranes. The membranes were blocked with 5% BSA in buffer containing 10 mM Tris-HCl, pH 7.5, 100 mM NaCl and 0.1% Tween 20. Once blocked, the membranes were probed with primary antibodies followed by horseradish peroxidase-conjugated antibody, and the blots were detected with enhanced chemiluminescence reagent (Dogen).

### Crude Synaptosome Fractionation

Mouse brain extracts were prepared as described [18], [46]. In brief, whole brains were homogenized in buffered sucrose containing 0.32M sucrose, 4 mM HEPES, pH 7.3, 1 mM MgCl_2_, 0.5 mM CaCl_2_, 10 mM NaF and 1 mM Na_3_VO_4_ supplemented with protease inhibitors. All centrifugation procedures were conducted at 4°C. The homogenate was centrifuged at 1,000 g for 10 min (yielding pellet P1). The supernatant (S1) was centrifuged at 12,000 g for 15 min (yielding supernatant S2). The resulting pellet was resuspended in buffered sucrose and centrifuged at 13,000 g for 15 min (yielding pellet P2; crude synaptosomes). For immunoprecipitation assays, the P2 fraction was extracted in modified RIPA buffer. For GST pull-down assays, it was extracted in DOC buffer containing 1% sodium deoxycholate and 50 mM Tris-HCl, pH 9.0.

### *In vitro* kinase Assays

Purified Src kinase (Upstate) was used for *in vitro* protein kinase assays with [γ-^32^P]-ATP and specific protein substrates. 20 mM PIPES, pH 7.5, 10 mM MnCl_2_, 10 mM dithiothreitol (DTT) and 5 U of purified Src was used in all assays. Src kinase was incubated with 2 µg of GST or GST-SPIN90 in 10 µl of 2x kinase buffer containing 5 µCi/µl [γ-^32^P]-ATP and 25 µM unlabeled ATP for 10 min at 30°C. Incubations were terminated by addition of sample buffer, after which the labeled products were resolved by SDS-PAGE and visualized by autoradiography.

### Phosphospecific Antibodies

Phosphospecific rabbit polyclonal antibody directed against SPIN90 phosphorylated on tyrosine 161 (anti-pY161) was generated using the phosphorylated synthetic KLH-conjugated peptide DGGL**pY**QIPLPC as antigen. The peptide was purchased from Anygen. The specific antibody was purified using a SulfoLink Immobilization Kit for Peptides (Thermo) and the immunogenic phosphorylated peptide followed by the corresponding unphosphorylated peptide.

### Statistical Analysis

Data were collected from at least three independent experiments carried out using independent neuronal cultures and then quantified. The statistical significance of differences between means was assessed using unpaired Student’s t tests. Data are presented as means **±** s.e.m. To evaluate the distribution of proteins between dendritic spines and shafts, spine and shaft fluorescence intensities were analyzed as ratios of the average fluorescence intensities in spines and adjacent dendritic shafts. SPIN90 intensity in spines was determined as the SPIN90 intensity in spines with both PSD95- and Vamp2-positive puncta. SPIN90 intensity in dendritic shafts was determined as the SPIN90 intensity in dendritic shafts corresponding to spines with both PSD95- and Vamp2-positive puncta. The measurements were analyzed using MetaMorph imaging software (Universal Imaging Cooperation). To examine spine lengths and widths, cells were co-transfected with RDF-actin to visualize the morphology of the dendritic spines in detail. To determine spine size, roughly 1000 spines (from 10–20 neurons) were measured under each condition. The heads of spines were measured by taking the maximal width of the spine head perpendicular to the axis along the spine neck. Spine length was measured as the distance from the base of the neck to the furthest point on the spine head. For each condition, individual spine dimensions were first grouped, and then averaged per neuron.

### Electrophysiology

Cultured hippocampal neurons (DIV 10–11) were transfected with GFP-SPIN90 WT or GFP-SPIN90 Y85, 161, 227E, GFP-SPIN90 Y85, 161, 227F or GFP alone. DIV 18–20 neurons were voltage clamped in the whole-cell configuration at a holding potential of −70 mV. The extracellular solution for voltage clamp experiments contained 150 mM NaCl, 4 mM KCl, 2 mM MgCl_2_, 2 mM CaCl_2_, 10 mM glucose and 10 mM HEPES (pH 7.4). To block spontaneous action potentials and mIPSCs during recording, 1 µM TTX (Tocris) and 40 µM bicuculline (Tocris) were added to the extracellular/recording solution. The pipette solution for mEPSC measurements contained 115 mM CsMeSO_3_, 10 mM CsCl, 5 mM NaCl, 10 mM HEPES, 10 mM EGTA, 4 mM Mg-ATP and 0.3 mM Na-GTP (pH 7.36). All recordings were made using an Axopatch 200 B amplifier (Axon instruments) at room temperature. Data were acquired at 10 kHz and low-pass filtered at 1 kHz. Pipette resistances were 2.5∼4.8 MΩ, and input resistance were >100 MΩ. The R_a_ (access resistance) value was constantly monitored in which recordings of cells exhibiting more than 15% change in the R_a_ value were discarded [47]. Upon whole-cell mode, whole-cell capacitance or membrane capacitance (C_m_) in pF and series resistance (R_s_) in MΩ were compensated and quantified via Axopatch 200 B and Clampfit software, respectively. Only those recordings with a series resistance below 30 MΩ were accepted, of which typical quantified data showed values less than 20 MΩ. Data were analyzed using Clampfit 6.1 (Axon Instruments) and presented using Origin 6.1 (OriginLab Corporation).

## References

[1] Bhatt DH, Zhang S, Gan WB (2009) Dendritic spine dynamics. Annu Rev Physiol 71: 261–282. 19575680 10.1146/annurev.physiol.010908.163140

[2] Hering H, Sheng M (2003) Activity-dependent redistribution and essential role of cortactin in dendritic spine morphogenesis. J Neurosci 23: 11759–11769. 14684878 10.1523/JNEUROSCI.23-37-11759.2003 PMC6740953

[3] Hori K, Yasuda H, Konno D, Maruoka H, Tsumoto T, et al (2005) NMDA receptor-dependent synaptic translocation of insulin receptor substrate p53 via protein kinase C signaling. J Neurosci 25: 2670–2681. 15758177 10.1523/JNEUROSCI.3638-04.2005 PMC6725157

[4] Wu Q, Dibona VL, Bernard LP, Zhang H (2012) The Polarity Protein Partitioning-Defective 1 (PAR-1) Regulates Dendritic Spine Morphogenesis through Phosphorylating Postsynaptic Density Protein 95 (PSD-95). J Biol Chem 287: 30781–30788. 22807451 10.1074/jbc.M112.351452 PMC3436321

[5] Li D, Specht CG, Waites CL, Butler-Munro C, Leal-Ortiz S, et al (2011) SAP97 directs NMDA receptor spine targeting and synaptic plasticity. J Physiol 589: 4491–4510. 21768261 10.1113/jphysiol.2011.215566 PMC3208220

[6] Ho GP, Selvakumar B, Mukai J, Hester LD, Wang Y, et al (2011) S-nitrosylation and S-palmitoylation reciprocally regulate synaptic targeting of PSD-95. Neuron 71: 131–141. 21745643 10.1016/j.neuron.2011.05.033 PMC3181141

[7] Chen L, Chetkovich DM, Petralia RS, Sweeney NT, Kawasaki Y, et al (2000) Stargazin regulates synaptic targeting of AMPA receptors by two distinct mechanisms. Nature 408: 936–943. 11140673 10.1038/35050030

[8] Schnell E, Sizemore M, Karimzadegan S, Chen L, Bredt DS, et al (2002) Direct interactions between PSD-95 and stargazin control synaptic AMPA receptor number. Proc Natl Acad Sci U S A 99: 13902–13907. 12359873 10.1073/pnas.172511199 PMC129795

[9] Cingolani LA, Goda Y (2008) Actin in action: the interplay between the actin cytoskeleton and synaptic efficacy. Nat Rev Neurosci 9: 344–356. 18425089 10.1038/nrn2373

[10] Chen Y, Wang F, Long H, Wu Z, Ma L (2011) GRK5 promotes F-actin bundling and targets bundles to membrane structures to control neuronal morphogenesis. J Cell Biol 194: 905–920. 21930777 10.1083/jcb.201104114 PMC3207290

[11] Nakamura Y, Wood CL, Patton AP, Jaafari N, Henley JM, et al (2011) PICK1 inhibition of the Arp2/3 complex controls dendritic spine size and synaptic plasticity. EMBO J 30: 719–730. 21252856 10.1038/emboj.2010.357 PMC3041953

[12] Lim CS, Park ES, Kim DJ, Song YH, Eom SH, et al (2001) SPIN90 (SH3 protein interacting with Nck, 90 kDa), an adaptor protein that is developmentally regulated during cardiac myocyte differentiation. J Biol Chem 276: 12871–12878. 11278500 10.1074/jbc.M009411200

[13] Kim DJ, Kim SH, Lim CS, Choi KY, Park CS, et al (2006) Interaction of SPIN90 with the Arp2/3 complex mediates lamellipodia and actin comet tail formation. J Biol Chem 281: 617–625. 16253999 10.1074/jbc.M504450200

[14] Satoh S, Tominaga T (2001) mDia-interacting protein acts downstream of Rho-mDia and modifies Src activation and stress fiber formation. J Biol Chem 276: 39290–39294. 11509578 10.1074/jbc.M107026200

[15] Teodorof C, Bae JI, Kim SM, Oh HJ, Kang YS, et al (2009) SPIN90-IRSp53 complex participates in Rac-induced membrane ruffling. Exp Cell Res 315: 2410–2419. 19460367 10.1016/j.yexcr.2009.05.010

[16] Lee S, Lee K, Hwang S, Kim SH, Song WK, et al (2006) SPIN90/WISH interacts with PSD-95 and regulates dendritic spinogenesis via an N-WASP-independent mechanism. EMBO J 25: 4983–4995. 16990791 10.1038/sj.emboj.7601349 PMC1618117

[17] Kim SM, Choi KY, Cho IH, Rhy JH, Kim SH, et al (2009) Regulation of dendritic spine morphology by SPIN90, a novel Shank binding partner. J Neurochem 109: 1106–1117. 19302483 10.1111/j.1471-4159.2009.06039.x

[18] Ko J, Kim S, Chung HS, Kim K, Han K, et al (2006) SALM synaptic cell adhesion-like molecules regulate the differentiation of excitatory synapses. Neuron 50: 233–245. 16630835 10.1016/j.neuron.2006.04.005

[19] Murata T, Ohnishi H, Okazawa H, Murata Y, Kusakari S, et al (2006) CD47 promotes neuronal development through Src- and FRG/Vav2-mediated activation of Rac and Cdc42. J Neurosci 26: 12397–12407. 17135401 10.1523/JNEUROSCI.3981-06.2006 PMC6674889

[20] Hotulainen P, Hoogenraad CC (2010) Actin in dendritic spines: connecting dynamics to function. J Cell Biol 189: 619–629. 20457765 10.1083/jcb.201003008 PMC2872912

[21] Frost NA, Kerr JM, Lu HE, Blanpied TA (2010) A network of networks: cytoskeletal control of compartmentalized function within dendritic spines. Curr Opin Neurobiol 20: 578–587. 20667710 10.1016/j.conb.2010.06.009 PMC2972359

[22] Zhao L, Ma QL, Calon F, Harris-White ME, Yang F, et al (2006) Role of p21-activated kinase pathway defects in the cognitive deficits of Alzheimer disease. Nat Neurosci 9: 234–242. 16415866 10.1038/nn1630

[23] Penzes P, Cahill ME, Jones KA, VanLeeuwen JE, Woolfrey KM (2011) Dendritic spine pathology in neuropsychiatric disorders. Nat Neurosci 14: 285–293. 21346746 10.1038/nn.2741 PMC3530413

[24] Raemaekers T, Peric A, Baatsen P, Sannerud R, Declerck I, et al (2012) ARF6-mediated endosomal transport of Telencephalin affects dendritic filopodia-to-spine maturation. EMBO J 31: 3252–3269. 22781129 10.1038/emboj.2012.182 PMC3411082

[25] Bingol B, Wang CF, Arnott D, Cheng D, Peng J, et al (2010) Autophosphorylated CaMKIIalpha acts as a scaffold to recruit proteasomes to dendritic spines. Cell 140: 567–578. 20178748 10.1016/j.cell.2010.01.024

[26] Bingol B, Schuman EM (2006) Activity-dependent dynamics and sequestration of proteasomes in dendritic spines. Nature 441: 1144–1148. 16810255 10.1038/nature04769

[27] Bramham CR, Wells DG (2007) Dendritic mRNA: transport, translation and function. Nat Rev Neurosci 8: 776–789. 17848965 10.1038/nrn2150

[28] Fujii R, Okabe S, Urushido T, Inoue K, Yoshimura A, et al (2005) The RNA binding protein TLS is translocated to dendritic spines by mGluR5 activation and regulates spine morphology. Curr Biol 15: 587–593. 15797031 10.1016/j.cub.2005.01.058

[29] Soderling SH, Guire ES, Kaech S, White J, Zhang F, et al (2007) A WAVE-1 and WRP signaling complex regulates spine density, synaptic plasticity, and memory. J Neurosci 27: 355–365. 17215396 10.1523/JNEUROSCI.3209-06.2006 PMC3740594

[30] Takahashi H, Sekino Y, Tanaka S, Mizui T, Kishi S, et al (2003) Drebrin-dependent actin clustering in dendritic filopodia governs synaptic targeting of postsynaptic density-95 and dendritic spine morphogenesis. J Neurosci 23: 6586–6595. 12878700 10.1523/JNEUROSCI.23-16-06586.2003 PMC6740629

[31] Terry-Lorenzo RT, Roadcap DW, Otsuka T, Blanpied TA, Zamorano PL, et al (2005) Neurabin/protein phosphatase-1 complex regulates dendritic spine morphogenesis and maturation. Mol Biol Cell 16: 2349–2362. 15743906 10.1091/mbc.E04-12-1054 PMC1087240

[32] Naisbitt S, Kim E, Tu JC, Xiao B, Sala C, et al (1999) Shank, a novel family of postsynaptic density proteins that binds to the NMDA receptor/PSD-95/GKAP complex and cortactin. Neuron 23: 569–582. 10433268 10.1016/s0896-6273(00)80809-0

[33] Pak DT, Yang S, Rudolph-Correia S, Kim E, Sheng M (2001) Regulation of dendritic spine morphology by SPAR, a PSD-95-associated RapGAP. Neuron 31: 289–303. 11502259 10.1016/s0896-6273(01)00355-5

[34] Hoe HS, Lee JY, Pak DT (2009) Combinatorial morphogenesis of dendritic spines and filopodia by SPAR and alpha-actinin2. Biochem Biophys Res Commun 384: 55–60. 19393616 10.1016/j.bbrc.2009.04.069 PMC2707853

[35] Choi J, Ko J, Racz B, Burette A, Lee JR, et al (2005) Regulation of dendritic spine morphogenesis by insulin receptor substrate 53, a downstream effector of Rac1 and Cdc42 small GTPases. J Neurosci 25: 869–879. 15673667 10.1523/JNEUROSCI.3212-04.2005 PMC6725612

[36] Lin YC, Koleske AJ (2010) Mechanisms of synapse and dendrite maintenance and their disruption in psychiatric and neurodegenerative disorders. Annu Rev Neurosci 33: 349–378. 20367247 10.1146/annurev-neuro-060909-153204 PMC3063389

[37] Saneyoshi T, Fortin DA, Soderling TR (2010) Regulation of spine and synapse formation by activity-dependent intracellular signaling pathways. Curr Opin Neurobiol 20: 108–115. 19896363 10.1016/j.conb.2009.09.013 PMC2856474

[38] Unoki T, Matsuda S, Kakegawa W, Van NT, Kohda K, et al (2012) NMDA receptor-mediated PIP5K activation to produce PI(4,5)P(2) is essential for AMPA receptor endocytosis during LTD. Neuron 73: 135–148. 22243752 10.1016/j.neuron.2011.09.034

[39] He K, Lee A, Song L, Kanold PO, Lee HK (2011) AMPA receptor subunit GluR1 (GluA1) serine-845 site is involved in synaptic depression but not in spine shrinkage associated with chemical long-term depression. J Neurophysiol 105: 1897–1907. 21307330 10.1152/jn.00913.2010 PMC3075297

[40] Bosch M, Hayashi Y (2012) Structural plasticity of dendritic spines. Curr Opin Neurobiol 22: 383–388. 21963169 10.1016/j.conb.2011.09.002 PMC4281347

[41] Kim SH, Choi HJ, Lee KW, Hong NH, Sung BH, et al (2006) Interaction of SPIN90 with syndapin is implicated in clathrin-mediated endocytic pathway in fibroblasts. Genes Cells 11: 1197–1211. 16999739 10.1111/j.1365-2443.2006.01008.x

[42] Kim Y, Kim S, Lee S, Kim SH, Park ZY, et al (2005) Interaction of SPIN90 with dynamin I and its participation in synaptic vesicle endocytosis. J Neurosci 25: 9515–9523. 16221862 10.1523/JNEUROSCI.1643-05.2005 PMC6725698

[43] Asrar S, Kaneko K, Takao K, Negandhi J, Matsui M, et al (2011) DIP/WISH deficiency enhances synaptic function and performance in the Barnes maze. Mol Brain 4: 39. 22018352 10.1186/1756-6606-4-39 PMC3208581

[44] Kim SM, Bae J, Cho IH, Choi KY, Park YJ, et al (2011) Control of growth cone motility and neurite outgrowth by SPIN90. Exp Cell Res 317: 2276–2287. 21763308 10.1016/j.yexcr.2011.06.018

[45] Kodama Y, Hu CD (2010) An improved bimolecular fluorescence complementation assay with a high signal-to-noise ratio. Biotechniques 49: 793–805. 21091444 10.2144/000113519

[46] Han K, Kim MH, Seeburg D, Seo J, Verpelli C, et al (2009) Regulated RalBP1 binding to RalA and PSD-95 controls AMPA receptor endocytosis and LTD. PLoS Biol 7: e1000187. 19823667 10.1371/journal.pbio.1000187 PMC2730530

[47] Sokolova IV, Lester HA, Davidson N (2006) Postsynaptic mechanisms are essential for forskolin-induced potentiation of synaptic transmission. J Neurophysiol 95: 2570–2579. 16394076 10.1152/jn.00617.2005

